# The efficacy of resilience training programs: a systematic review protocol

**DOI:** 10.1186/2046-4053-3-20

**Published:** 2014-03-06

**Authors:** Aaron L Leppin, Michael R Gionfriddo, Amit Sood, Victor M Montori, Patricia J Erwin, Claudia Zeballos-Palacios, Pavithra R Bora, Megan M Dulohery, Juan Pablo Brito, Kasey R Boehmer, Jon C Tilburt

**Affiliations:** 1 Knowledge and Evaluation Research Unit, Mayo Clinic, 200 First Street SW, Rochester, MN 55905, USA; 2 Mayo Graduate School, Mayo Clinic, 200 First Street SW, Rochester, MN 55905, USA; 3 Biomedical Ethics Research Unit, Mayo Clinic, 200 First Street SW, Rochester, MN 55905, USA; 4 Division of General Internal Medicine, Mayo Clinic, 200 First Street SW, Rochester, MN 55905, USA; 5 Division of Endocrinology, Metabolism, and Nutrition, Mayo Clinic, 200 First Street SW, Rochester, MN 55905, USA; 6 Division of Pulmonary and Critical Care, Mayo Clinic, 200 First Street SW, Rochester, MN 55905, USA; 7 Mayo Clinic Libraries, 200 First Street SW, Rochester, MN 55905, USA; 8 Rochester, MN 55905, USA

**Keywords:** Resilience, Resilience training, Systematic review, Randomized controlled trials

## Abstract

**Background:**

Resilience has been defined as the ability of individuals to manage and adapt to stress and life challenges. Training programs that develop and/or enhance resilience may have efficacy in improving health, well-being, and quality of life. Because patients with chronic conditions must reliably self-manage their health, strategies to bolster resilience in this population may be of particular value. The objectives of this systematic review are to synthesize the evidence of resilience training program efficacy in improving outcomes related to quality of life, self-efficacy and activation, and resilience and coping ability in: 1) diverse adult populations; and 2) patients with chronic conditions.

**Methods/Design:**

We will conduct a systematic review of randomized controlled trials assessing the efficacy of any program designed to enhance resilience in adults that measure any outcome against any comparator. We will search multiple electronic databases, trial registries, bibliographies, and will contact authors and experts to identify studies. We will use systematic review software to independently and in duplicate screen reports and extract data. We will extract characteristics of the study populations, interventions, comparators, outcomes, and quality/risk of bias. Primary, patient reported outcomes will be categorized into domains of quality of life, self-efficacy, and resilience. Secondary outcomes will be considered based on findings of the review. We will attempt meta-analysis by pooling standardized mean differences and minimally important differences (MIDs), when possible. Planned trial subgroup analyses are: 1) studies of patients with chronic conditions; 2) studies with placebo controls; 3) studies with similar intervention characteristics; and 4) studies with common lengths of follow-up.

**Discussion:**

This study is intended to accumulate the evidence for resilience training programs in improving quality of life, resilience, and self-efficacy for care management, particularly among adult patients with chronic conditions. Its findings will be valuable to policy-makers, funding agencies, clinicians, and patients seeking innovative and effective ways to achieve patient-centered care.

**Trial registration:**

PROSPERO registration number: CRD42014007185.

## Background

Resilience has been defined as ‘the process of negotiating, managing, and adapting to significant sources of stress or trauma’ [1] and as ‘the ability of an individual to adjust to adversity, maintain equilibrium … and continue to move on in a positive manner’ [2]. Resilient persons often exhibit characteristics of commitment, dynamism, humor in the face of adversity, patience, optimism, faith, and altruism [2]. Although perhaps traditionally thought of as an intrinsic, non-modifiable human attribute, evidence suggests that resilience may be both a ‘movable’ and teachable construct [3-6]. It also appears to be heavily influenced by external and modifiable social factors [1,7]. International interest in resilience research has increased in recent years and a number of stakeholders are considering the role resilience plays in influencing health, well-being, and quality of life [1,8,9]. Because patient self-perception of health and well-being is an important predictor of many relevant outcomes [10,11], improving personal resilience may offer new and effective approaches to care delivery [3,12,13].

In chronic conditions, patients must reliably self-manage their care. The ability of patients to carry out this work is affected by a number of inter-related personal patient characteristics. These include activation [14], self-efficacy [15], and resilience [13]. Identifying effective ways to promote the development of these characteristics in this population can improve coping ability and prepare patients for the long and unpredictable journey of living with a chronic condition [16-19].

Training programs that develop and/or enhance resilience vary in structure and format and have been tried in a variety of populations. Mediating variables that are commonly addressed in these programs include the promotion of positive emotions, cognitive flexibility, social support, life meaning, and active coping [20]. Unlike mindfulness-based stress reduction (MBSR) or other meditation-based programs, which primarily seek to address active issues of stress and/or anxiety [21], resilience-specific training (as currently conceptualized) is primarily forward-looking (for example ‘preventive’) [3] and seeks to foster personal qualities needed to deal with unanticipated stressors. To our knowledge, no systematic review of the efficacy of resilience training programs exists. To better understand the potential for resilience training to bolster patient self-management capacity in chronic conditions, we will examine the literature on this topic.

The objectives of this proposed systematic review are to:

1) Synthesize the evidence of resilience training program efficacy in improving outcomes related to quality of life, self-efficacy and activation, and resilience and coping ability in diverse adult populations.

2) Estimate the aggregate efficacy of resilience training programs in improving quality of life, self-efficacy and activation, and resilience and coping ability among patients with chronic conditions.

## Methods/Design

### Study design

We will conduct a systematic review adhering to the reporting guidelines of the preferred reporting items for systematic reviews and meta-analyses (PRISMA) statement [22].

### Study registration

This systematic review is registered with PROSPERO (registration number: CRD42014007185; http://www.crd.york.ac.uk/PROSPERO).

### Criteria for considering studies for this review

#### Type of studies

We will include randomized controlled trials published within or after the year 1990 that evaluate the efficacy of any program specifically designed to develop or enhance subject resilience. We will exclude historically controlled, quasi-experimental, and single-arm pre-post studies.

#### Type of participants

We will include studies of all adults (≥18 years old). Studies of children will be excluded.

#### Type of interventions

Eligible interventions will include any program specifically designed to develop or enhance subject resilience, as determined by a consensus of the authors. These programs may have additional elements (MBSR, meditation, yoga, for example) but cannot be solely focused on stress-reduction or ‘reactive’ methods. Similarly, although many interventions are likely to affect resilience secondarily, we will restrict our inclusion only to those interventions that prospectively and systematically aim to impact this construct primarily. Such interventions should generally describe a theoretical or scientific rationale for why the intervention would be expected to impact resilience.

#### Type of outcome measures

We are primarily interested in patient reported outcomes that measure one or more of the following three construct domains: 1) quality of life or well-being; 2) activation or self-efficacy; and 3) resilience or ability to cope. Other secondary outcomes will be considered, depending on what is uncovered during the review; anticipated possibilities include depression, stress, and anxiety.

### Search methods for the identification of studies

We will design and conduct a search strategy using methods recommended by the Institute of Medicine [23]. This will involve the searching of several electronic databases: PubMed, Scopus, EBSCO CINAHL, Ovid MEDLINE, Ovid EMBASE, Ovid Cochrane Library, Web of Science, and Ovid PsycINFO. Because resilience itself is an evolving construct [3,24,25] and the concept of resilience training is still relatively new, being continuously informed by scientific advances, and unlikely to have been operationalized in similar ways many years ago [3], databases will be searched only from January 1, 1990 to the current time. To maximize the specificity and feasibility of the search, we will focus on Medical Subject Headings (MeSH) terms such as ‘resilience, psychological’ and ‘randomized controlled trials’ along with text searches of ‘resilience’ and ‘hardiness’ (which is a term used by some in referencing a similar construct), among others. The concept of training will be addressed primarily using textwords such as ‘train,’ ‘teach,’ and ‘skills.’ The initial electronic search strategy will be supplemented by hand searching the reference lists from eligible included studies and through contacting experts in the field to identify any missing, in-progress, or unpublished studies. In addition, we will search for reviews on the topic and search through their reference lists to identify any potentially eligible studies that may have been missed through other methods. Finally, clinical trial registries will be searched to identify completed and in-progress studies and, if not identified through other methods, the authors will be contacted for details regarding the study’s status. There will be no language restrictions during the initial search.

### Selection of studies

We will upload search results into systematic review software (DistillerSR, Ottawa, ON, Canada). In the first round of screening, abstracts and titles will be screened for inclusion. Following abstract screening, eligibility will be assessed through full-text screening. Prior to both abstract and full-text screening, reviewers will undergo training to ensure a basic understanding of the background of the field and purpose of the review. Comprehension of the inclusion and exclusion criteria will also be assessed through calibration on a small number of studies. Eligibility at both levels (abstract and full-text) will be assessed independently and in duplicate. Any disagreements will be resolved by consensus. If consensus cannot be achieved between the two reviewers, a third reviewer will arbitrate.

### Data extraction

Data extractors, working independently, will collect primary data from the included trials through use of a web-based program (DistillerSR). The extracted data will include patient characteristics, outcomes measured and instruments used, intervention and control characteristics, and factors associated with study quality. Discrepancies in data collection will be adjudicated by consensus.

If data presented in the studies is unclear, missing, or presented in a form that is either un-extractable or difficult to reliably extract, the authors of the study will be contacted for clarification. When data extraction is complete, the authors of the studies will be contacted to ensure the accuracy and completeness of the data extraction. In addition, at this time the authors of included studies will be asked if they know of any additional studies, either completed or ongoing, that they believe would be eligible for our review.

Author contact will be initiated by an email to the corresponding author. If an email is unavailable an internet search will be used to find a current email address; when emails are available, first authors of manuscripts will be carbon copied on all emails to the corresponding author. If emails for the corresponding author are unavailable, corresponding authors will be contacted by telephone. Authors will be given a week to respond to emails, at which time a follow-up email will be sent, if no response is received after an additional two weeks a telephone call will be made to try to contact the author. Attempts to reach authors by telephone will occur throughout the week for a period of two weeks at which time the author will be classified as un-contactable.

### Risk of bias assessment

We will use the Cochrane Collaboration’s risk of bias tool [26] to evaluate the methodological quality of included studies. The risk of bias (high/low/unclear) in included studies will be assessed in duplicate by reviewers working independently. Any disagreements will be resolved by consensus; if consensus is unable to be achieved a third reviewer will arbitrate. Items included in the risk of bias assessment will include: randomization, quality of randomization (any important imbalances at baseline), allocation concealment, level(s) of blinding/masking, losses to follow-up, intention to treat analysis, handling of missing data, and funding sources.

### Analysis

We will first summarize and describe the populations, interventions, and outcomes studied. Descriptive statistics will be used as appropriate to compare the characteristics of the studies and narratives will be used as necessary to describe the interventions. The patient reported outcomes of the included studies will be categorized by construct similarity into the following domains: 1) quality of life or well-being; 2) activation or self-efficacy; and 3) resilience or ability to cope. This process will involve retrieval of the published description of each instrument’s construction and validation and/or a relevant review of instruments. Domain categorization will be based on consensus among the authors. To deal with the potential scenario in which multiple outcomes and/or measures are used within a single domain for a given study, we will develop an outcome hierarchy to determine which outcome to extract for analysis. This hierarchy will prioritize outcomes that are: a) commonly reported; b) highly validated and/or have a determined minimally important difference (MID); and c) endorsed by the National Quality Forum [27]. Additional, secondary outcomes will be considered based on what the review uncovers. For any part of this systematic review where subjective assessment is required, the agreement between two reviewers will be measured using kappa or phi statistics, as appropriate [28] (the latter is appropriate when the distribution of agreement is extreme).

#### Summary measures

We will conduct a meta-analysis of pooled standardized mean differences (SMDs) as suggested by the *Cochrane Handbook for Systematic Reviews of Interventions*[29] and additionally report, if possible, in terms of MIDs, as recommended by Johnston *et al*. [30,31]. Alternatively, if the included studies consistently use a single, common instrument, we will consider converting outcomes from other instruments to the common instrument’s natural units and report the pooled effect in this way [30]. We will report both random and fixed effects models in the case of a discrepancy between them; otherwise, we will report the random effects model only. We will measure inconsistency for each outcome by estimating the I^2^ test and its associated confidence interval [32]. We will use RevMan software [33] to conduct the analyses.

#### Missing data

If missing data exists within the included trials, we will contact the authors to see if it is obtainable. If the data is unobtainable, we will use the complete case analysis and conduct sensitivity analysis for continuous outcomes and dichotomous outcomes using the methods of Ebrahim *et al*. [34] and Akl *et al*. [35], respectively.

#### Risk of bias across studies

Publication bias will be assessed by plotting the estimate of effect of trials by the inverse of its standard error using a funnel plot. The plots will be assessed both visually and by using Egger’s test [36]; a significant publication bias will be considered to exist if the *P* value is <0.1.

#### Additional analyses

Following the primary analyses, several *a priori*, exploratory sub-group analyses will be performed. These include stratification by: 1) whether the patient population had a chronic condition (defined as any condition expected to last at least one year and require ongoing management); 2) the type of comparator used (that is, placebo/sham versus usual care); 3) the intervention structure or format; 4) the length of follow-up; and 5) the quality of the study/risk of bias. We may perform additional subgroup analyses if the search reveals large numbers of studies in certain populations (for example, healthcare workers). If intervention structure proves to be highly variable and the number of studies is sufficiently large, we may attempt to explain variation in effects by conducting a meta-regression analysis of intervention characteristics.

## Discussion

Healthcare should seek to prioritize outcomes that matter to patients [27]. In many cases, this may mean the pursuit of patient goals for life and health at the expense of metabolic parameters or guideline-based protocols. This subtle paradigm shift in care delivery requires a rethinking of the sorts of strategies that are most likely to be useful. Resilience training programs are an intriguing intervention that may have value in this regard, but no high-quality synthesis of the evidence exists.

This study will face a number of limitations that may limit its ability to generate conclusions based on high confidence. Specifically, there may be significant heterogeneity in the populations studied and the structure and theoretical approaches of interventions tried. There will also likely be differences in the outcomes measured and tools used. Pooling this data within construct domains carries inherent uncertainty.

We have also elected to exclude studies in children, which will limit the applicability of our findings to this population. Although many studies of resilience have been conducted in children, these have largely sought to understand the antecedents and predictors of resiliency. The question we seek to answer, however, is more concerned with determining the usefulness of interventions to promote resiliency; we anticipate that such interventions, in clinical practice, will more frequently be tried in adults.

## Conclusion

This study is intended to accumulate the evidence for resilience training programs in improving quality of life, resilience, and self-efficacy for care management, particularly among adult patients with chronic conditions. Its findings will be valuable to policy-makers, funding agencies, clinicians, and patients seeking innovative and effective ways to achieve patient-centered care.

## Abbreviations

MBSR: Mindfulness-based stress reduction; MeSH: Medical Subject Headings; MID: Minimally important difference; PRISMA: Preferred reporting items for systematic reviews and meta-analyses; SMD: Standardized mean difference.

## Competing interests

One of the authors, AS, has published extensively on this topic and has written a book about resilience. AS did not conceptualize this project or any part of its approach, but was sought out for expertise and direction.

## Authors’ contributions

AL, JT, VM, AS, PE, CZ, BR, KB, MG, PB, MD, and JB conceived and designed the study, and wrote the manuscript. All authors read and approved the final manuscript.
